# Clinical and structural outcomes after arthroscopic rotator cuff repair: a comparison between suture bridge techniques with or without medial knot tying

**DOI:** 10.1186/s13018-018-0990-z

**Published:** 2018-11-22

**Authors:** Hirokazu Honda, Masafumi Gotoh, Yasuhiro Mitsui, Hidehiro Nakamura, Ryo Tanesue, Hisao Shimokobe, Naoto Shiba

**Affiliations:** 1 Department of Orthopaedic Surgery, Kurume University Medical Center, 155-1 Kokubu-machi, Kurume, 839-0863 Japan; 2 Department of Orthopaedic Surgery, Kurume University Hospital, 67 Asahi-machi, Kurume, Japan

**Keywords:** Arthroscopic rotator cuff repair, Suture bridge, Medial tying

## Abstract

**Purpose:**

To compare arthroscopic suture bridge (SB) techniques with medial tying to those without tying, considering clinical and structural outcomes.

**Methods:**

We included 124 patients with rotator cuff tears after arthroscopic rotator cuff repair (ARCR). Fifty-three patients with clinical and structural evaluations 3, 12, and 24 months postoperatively were included and divided into 29 patients with medial tying (WMT group) and 24 without tying (WOMT group). Clinical outcomes comprised the University of California Los Angeles (UCLA) and Japanese Orthopaedic Association (JOA) scores. Structural outcomes were evaluated with magnetic resonance images (MRI) using Sugaya classifications.

**Results:**

JOA and UCLA scores in the WMT and WOMT groups improved significantly from before surgery to 24 months after surgery (*P* < 0.01, respectively). No significant difference was noted between groups. No significant postoperative retears (Sugaya types 4 and 5) between WMT and WOMT groups were noted at 3 months (5 vs 3 cases), 12 months (6 vs 5 cases), and 24 months (7 vs 6 cases) postoperatively. Complete healing (Sugaya type 1) was noted at 3 months (8 vs 11 cases), 12 months (10 vs 10 cases), and 24 months (8 vs 13 cases, *P* = 0.024) postoperatively. Incomplete healing (Sugaya types 2 and 3) were noted at 3 months (16 vs 10 cases), 12 months (13 vs 9 cases), and 24 months (14 vs 5 cases, *P* = 0.024) postoperatively.

**Conclusion:**

Clinical outcomes for both techniques were comparable, but the number of incompletely healed tendons in SB with medial tying was significantly larger at 24 months after surgery.

**Level of evidence:**

This study is a level III, case-control study.

**Clinical relevance:**

This study revealed the influence of medial tying in rotator cuff repair.

## Introduction

The prevalence of rotator cuff tears ranges between 5 and 30%, occurring approximately 50% of the time in adults aged 70 years or more [14, 17]. When conservative therapy is not effective, surgical treatment is performed. Arthroscopic rotator cuff repair (ARCR) is known to be a successful procedure that restores function and provides satisfactory pain relief. One of the most serious matters after surgery is the retear rate. Despite advances in surgical techniques, retears occur at rates of 11% to 57% [9].

To obtain high initial fixation, large contact area with footprint, and mechanical stability until tendon-bone healing [5], a suture bridge technique is used in arthroscopic rotator cuff repairs. In this technique, knot tying in a medial row is often performed because of its biomechanical advantage [14, 21]. On the contrary, a basic study pointed out the disadvantages of this process evidenced by strangulation due to the medial tying [14, 18].

Few studies have compared the clinical outcomes of the suture bridge technique with or without medical tying [1], although clinical differences were not demonstrated between these techniques, in terms of functional and structural outcomes. Therefore, the purpose of this study was to compare the functional and structural outcomes in patients with rotator cuff tears who underwent ARCR using suture bridge techniques with or without medal tying. We hypothesized that no significant difference in clinical outcome would be noted, but that there would be a significant difference in postoperative cuff integrity between the two techniques.

## Methods

The study details were thoroughly explained to the participants, each of whom provided consent to participate and publication. This study was approved by the institutional review board of our institute and reported as retrospective study (# 13306).

### Patient selection

Between July 2011 and December 2013, 124 patients with rotator cuff tears underwent ARCR by a suture-bridging (SB) technique in our institute. The inclusion criteria were (1) patients with full-thickness cuff tears and (2) those with both clinical evaluations and magnetic resonance images (MRI) at 3, 12, and 24 months after surgery. Patients were excluded if they had partial repairs, open repairs, revision surgeries, fractures, osteoarthritis of the glenohumeral joint, any rheumatic condition, or neurological involvement. Consequently, 53 patients (53 shoulders) with the average age of 63.8 ± 9.2 years were included in this study.

### Surgical procedures

Under general anesthesia, the patients were placed in the beach-chair position. A posterior portal was first established for the initial evaluation of the glenohumeral joint. During the examination, the tear location and size, delamination, and associated biceps tendon lesions were inspected carefully. The associated biceps tendon lesion was treated by tenotomy. Then, the arthroscope was removed from the glenohumeral joint and redirected into the subacromial space. Bursal tissue was removed for space clearance, and arthroscopic subacromial decompression was routinely performed to smooth the acromial undersurface in all patients. The footprint was prepared by removing the soft tissue and bony abrasions with an arthroscopic bur, preserving the cortical rim.

**Fig. 1:**
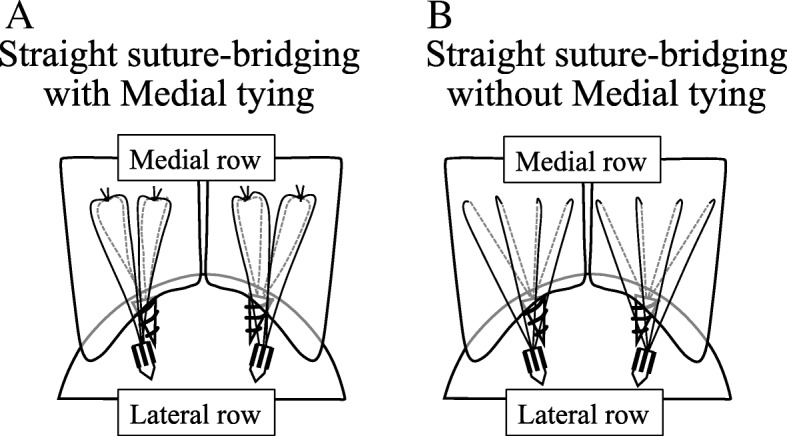
**a** Schematic showing the surgical procedures with medial knot tying. **b** Schematic showing the surgical procedures without medial knot tying

For the suture-bridging with medial tying (Fig. 1a), medial row anchors (Panalock RC: 4.7 mm × 11 mm, DePuy Mitek, Raynham, MA) that were double-loaded (four strands: 0.5-mm diameter wire, FiberWire, Arthrex) were inserted just lateral to the cartilage of the humeral head. The number of medial row anchors placed depended on the tear size. A suture from each anchor was passed through the tendon with a Scorpion Suture Passer (DePuy Mitek, Raynham, MA). This procedure was repeated for the remaining sutures. A horizontal mattress configuration was created with a 1-cm interval between each of the mattress stitches. After tying the knots without cutting the wires, pilot holes were prepared for the knotless, laterally inserted anchors (Versalock, DePuy Mitek, Raynham, MA) that were to be placed approximately 5–10 mm distal to the lateral edge of the greater tuberosity. At this step, four wire strands from 1 anchor were retrieved through a portal. These wire strands were threaded through the eyelet on the distal end of the lateral anchor. With the tendon reduced to a suitable position on the footprint, the anchor was inserted, thereafter adjusting the tension of the tendon tissue. The procedure was repeated to place the second lateral anchor for the suture bridge repair.

For the procedure without knot tying, arthroscopic, knotless, self-reinforcing, suture bridge repairs were performed in the same manner (Fig. 1b).

### Postoperative protocol

All patients followed the same rehabilitation regimen. The shoulders were immobilized for 6 weeks in a sling with an abduction pillow. Range of motion (ROM) exercises for the elbow, wrist, and fingers were started immediately after surgery. Passive forward elevation exercises were initiated from postoperative day 1. At 4 weeks after surgery, active-assisted motion exercise began, and at 6 weeks, active motion commenced. Eight weeks after surgery, a strengthening exercise program was allowed.

### Clinical evaluation

The clinical assessment consisted of the Japanese Orthopaedic Association (JOA) and University of California, Los Angeles (UCLA) scoring systems. These outcome measures were evaluated preoperatively and postoperatively at 3, 12, and 24 months.

### Structural evaluation

The rotator cuff integrity was determined by using magnetic resonance images (MRI; 1.5-Tesla [Excelart; Toshiba Medical Systems, Tokyo, Japan]) obtained preoperatively, at 6, 12, and 24 months after surgery. Any tendon defect filled with fluid was considered a tear [8, 13, 16]. Postoperative rotator cuff integrity was classified into five categories using the Sugaya classification [20]: type 1 indicated a completely healed tendon, types 2 and 3 indicated incompletely healed tendon, and types 4 and 5 indicated a re-torn tendon after surgery.

In cases of retear, according to the patterns reported by Cho et al. [3], retears were classified into type 1 (unhealed tendon) and type 2 (medially ruptured tendons) with a healed foot print.

### Statistical analysis

The software JMP11 (SAS Institute, Cary, NC, USA) was used for statistical analysis. The Mann-Whitney *U* test was used to compare the continuous and nominal variables in the patients’ demographic data and the JOA/UCLA scores between the two groups. One-way analysis of variance (ANOVA) was used to compare the JOA and UCLA scores before and after surgery. The chi-square test was used to evaluate postoperative tendon integrity (complete/incomplete healed, or retear) between the with medial tying (WMT) and without medial tying (WOMT) groups. A *P* value less than 0.05 was considered statistically significant.

## Results

The subjects were divided into two groups: 29 shoulders treated by the SB technique with medial tying (WMT group with an average age of 63.8 ± 8.4 years) and 24 shoulders without medial tying (WOMT group with an average age of 65.1 ± 9.6 years). The size of cuff tears (WMT vs WOMT) was 2 and 3 cases in small tears, 9 and 6 cases in middle tears, 12 and 9 in large tears, and 6 and 6 cases in massive tears; consequently, no significant difference of the tear size was noted between the two groups. The demographic data of each group are shown in Table 1.

**Table 1:** Demographic data of patients with (WMT group) or without (WOMT group) medial knot tying during surgery

	WMT (*n* = 29)	WOMT (*n* = 24)	*P* value
Age, year	63.8 ± 8.4	65.1 ± 9.6	0.70
Gender, *n*			
Male	17	15	
Female	12	9	0.77
Traumatic onset, *n*	15	12	0.90
Symptom duration, week	35.5 ± 25.2	33.5 ± 29.1	0.55
Tear size, *n*			
Small	2	3	
Middle	9	6	
Large	12	9	
Massive	6	6	0.86
ROM, degree			
Flex	114.2 ± 39.7	111.0 ± 29.4	0.70
Abd	103.2 ± 49.3	101.7 ± 41.1	0.81
BR	4.7 ± 3.5	4.0 ± 3.0	0.40
ER	42.0 ± 19.4	39.0 ± 13.3	0.88
MS			
Flex	110.7 ± 119.4	99.8 ± 25.6	0.17
Abd	59.3 ± 25.5	65.9 ± 30.0	0.59
IR	87.1 ± 25.5	102.2 ± 30.4	0.16
ER	80.5 ± 29.2	72.4 ± 21.0	0.55

*n* number, *ROM* range of motion, *Flex* flexion, *Abd* abduction, *BR* back reach, *MS* muscle strength, *ER* external rotation, *IR* internal rotation

### Clinical outcomes

JOA score in the WMT and WOMT groups improved significantly from 66.6 ± 14.5 and 62.6 ± 13.5 points before surgery, respectively, to 74.2 ± 19.2 and 74.5 ± 1.38 points at 3 months (*P* < 0.01), 89.6 ± 8.5 and 86.0 ± 12.3 points at 12 months (*P* < 0.01), and 94.2 ± 6.0 and 87.8 ± 12.9 points at 24 months after surgery (*P* < 0.01). No significant differences were noted between both groups for each phase evaluated.

Similarly, the UCLA scores improved significantly from 17.5 ± 5.3 and 16.7 ± 3.1 points before surgery, respectively, to 27.1 ± 3.3 and 27.7 ± 3.2 points at 3 months (*P* < 0.01), 32.6 ± 2.2 and 30.2 ± 5.4 points at 12 months (*P* < 0.01), and 34.6 ± 12.8 and 33.4 ± 3.4 points at 24 months after surgery (*P* < 0.01). No significant differences were noted between both groups for each phase evaluated, and the details are shown in Table 2.

**Table 2:** Clinical outcome in patients with (WMT group) or without (WOMT group) medial knot tying during surgery

	WMT (*n* = 29)	WOMT (*n* = 24)	*P* value
JOA score			
BO	66.6 ± 14.5	62.6 ± 13.5	0.37
PO 3M	74.2 ± 19.2	74.5 ± 13.8	0.69
PO 1Y	89.6 ± 8.5	86.0 ± 12.3	0.41
PO 2Y	94.2 ± 6.0	87.8 ± 12.9	0.21
UCLA score			
BO	17.5 ± 5.3	16.7 ± 3.1	0.56
PO 3M	27.1 ± 3.3	27.7 ± 3.2	0.27
PO 1Y	32.6 ± 2.2	30.2 ± 5.4	0.42
PO 2Y	34.6 ± 2.8	33.4 ± 3.4	0.52

*BO* before operative, *PO* Postoperative, *M* month, *Y* year, *JOA* Japan Orthopedic Association, *UCLA* University of California, Los Angeles

### Structural outcome: retear cases

Postoperative retears (Sugaya types 4 and 5) in the WMT and WOMT groups were noted, respectively, in 5 and 3 cases at 3 months after surgery (*P* = 0.63), 6 and 5 cases at 12 months after surgery (*P* = 0.99), and 7 and 6 cases at 24 months after surgery (*P* = 0.94). There were no significant differences between the two groups in each phase evaluated. According to Cho’s classification, all of these retear cases consistently showed a type 2 retear pattern. These details are shown in Table 3.

**Table 3:** Number of retear in patients with (WMT group) or without (WOMT group) medial knot tying during surgery

	WMT (*n* = 29)	WOMT (*n* = 24)	*P* value
Retear			
PO3M	5	3	0.63
PO1Y	6	5	0.99
PO2Y	7	6	0.94

*PO* postoperative, *M* month, *Y* year

### Structural outcome: Completely or incompletely healed cases

Type A cases, classified as completely healed (Sugaya type 1), in the WMT and WOMT groups was noted, respectively, in 8 and 11 cases at 3 months after surgery (*P* = 0.197), 10 and 10 cases at 12 months after surgery (*P* = 0.554) 8 and 13 cases at 24 months after surgery (*P* = 0.024). There were no significant differences between the two groups in each phase evaluated.

Type B cases, classified as incompletely healed (Sugaya types 2 and 3), in the WMT and WOMT groups was noted, respectively, 16 and 10 cases at 3 months after surgery (*P* = 0.197), 13 and 9 cases at 12 months after surgery (*P* = 0.554), 14 and 5 cases at 24 months after surgery (*P* = 0.024). The number of type B cases was not significantly different at 3 or 12 months after surgery, but the number of cases in the WMT group at 24 months after surgery was significantly greater than those in the WOMT group (*P* = 0.024). These details are shown in Table 4.

**Table 4:** Number of complete (Sugaya type 1) and incomplete (Sugaya types 2 and 3) healing in patients with (WMT group) or without (WOMT group) medial knot tying during surgery

	WMT	WOMT	*P* value
PO3M			
Complete healing	8	11	0.20
Incomplete healing	16	10	
PO1Y			
Complete healing	10	10	0.55
Incomplete healing	13	9	
PO2Y			
Complete healing	8	13	0.02
Incomplete healing	14	5	

*PO* postoperative, *M* month, *Y* year

## Discussion

The suture bridge technique in ARCR is associated with good clinical outcomes [7, 14], although the effect of the medial knot tying on postoperative structural outcome with this technique remains controversial. The present study compared the clinical and structural outcomes between the techniques with or without knot tying, at 3, 12, and 24 months after surgery. We found that there were no significant differences in clinical outcomes and retear rates after surgery throughout the periods. However, incomplete healing increased significantly in patients with knot tying at 24 months after surgery, compared with in those without tying. To our knowledge, no previous studies have shown such data.

Most biomechanical studies support the advantage of medial knot in the suture bridge technique. Leek et al. reported that the creation of medial knots increases construct stiffness and stability in the double-row, transtendon repair [11]. Busfield et al. demonstrated that although lateral row knotless fixation has been shown not to sacrifice the structural integrity of this construct, the addition of a knotless medial row compromises the construct leading to greater gapping and failure at lower loads [2]. In contrast, Sano et al. revealed that higher stress concentrations exist around the medial anchor in the double-row fixation, suggesting a biomechanical disadvantage of the medial knot tying [18]. Taken together, these reports suggest that although medial knot tying offers a biomechanical advantage, a high stress concentration at the tying site affects tendon integrity after surgery.

Another clinical study examined the effect of medial tying in the suture bridge technique on clinical outcomes after ARCR. Hayashida et al. examined the retear pattern after arthroscopic double-row repair and showed that complete tearing around the medial-row anchors with a well-repaired tendon could be characteristic of this procedure [6]. Similarly, Kim et al. [10] and Cho et al. [3] reported that retear patterns in the suture bridge technique with medial knot tying are predominantly associated with medially ruptured tendons with a healed foot print. These studies consistently emphasized the importance of medial anchors with knot tying in the suture bridge technique; however, the advantage of a knotless medial anchor was not demonstrated.

Boyer et al. [1] compared the suture bridge with medial tying (group A) with knotless bridging using suture tape (group B). In their study, both techniques achieved successful functional outcomes. The retear rate tended to be higher in group A (23.4%) than in group B (17.1%), but the difference did not reach statistical significance. The present study revealed similar results; however, the number of incompletely healed tendons at the final follow-up was significantly greater in the WMT group than in the WOMT group. Unlike the medial-tying suture bridge, the knotless suture bridge not only restored the pressured footprint, but also reduced the tension overload of the suture-tendon interface in the medial row [18, 19]. This reduces the possibility of strangulation and subsequent necrosis of the tendon at the medial row [4, 12]. Thus, these effects may explain why the knotless bridging in the medial row was superior to the knotted technique in terms of structural outcomes.

Medial failure is often observed in the medial knot-tying suture bridge, where re-rupture does not occur at the original repair site, but occurs more medially. Kim et al. compared the retear pattern of three repair methods, including the single row (SR), the medial-tying suture bridge (SB), and the knotless (SB), and concluded that the retear patterns in medial-tying SB (predominantly showing type 2) was different from that of SR (predominantly showing type 1) [3]. This finding is consistent with the report of Cho et al. [10]. In the present study, the retears consistently exhibited a type 2 pattern, suggesting that stress concentrates at the medial row in both techniques. Suture bridges were usually constructed by a “cross form,” and not by a “straight form” as used in the present study. Thus, this discrepancy would have led to the outcomes obtained from the present study.

There were several limitations of the present study. First, the present study was retrospective and had a small sample size. Second, whether or not the repaired tendons of Sugaya types 2 and 3 were incompletely healed was not elucidated experimentally. However, since normal rotator cuff tendons usually depict a homogeneous low-intensity on MR images [15], we believe that our interpretation (type 1 as completely healed and types 2 and 3 as incompletely healed) is valid and conceivable.

Few studies have compared the clinical outcomes of the suture bridge technique with or without medical tying [1], although clinical differences were not demonstrated between these techniques, in terms of functional and structural outcomes. On the contrary, a strong point of this study was that we sequentially observed the cuff integrity at 3, 12, and 24 months after surgery, which disclosed the statistical differences in structural outcomes between the patients based on the two techniques, showing the superiority of the technique without medial tying.

## Conclusions

The clinical outcomes for both techniques were comparable throughout the periods evaluated, but the number of incompletely healed tendons after SB with medial knot tying was significantly increased at 24 months after surgery, compared to those occurring after SB without medial knot tying.

## Abbreviations

ARCR: Arthroscopic rotator cuff repair
JOA: Japanese Orthopedic Association
MRI: Magnetic resonance imaging
ROM: Range of motion
SB: Suture bridging
UCLA: University of California Los Angeles
WMT: With medial tying
WOMT: Without medial tying
